# Pentamethylquercetin Attenuates Cardiac Remodeling via Activation of the Sestrins/Keap1/Nrf2 Pathway in MSG-Induced Obese Mice

**DOI:** 10.1155/2020/3243906

**Published:** 2020-01-30

**Authors:** Jingxia Du, Wei He, Cai Zhang, Jianzhao Wu, Zhi Li, Min Wang, Shuying Feng, Gaofeng Liang

**Affiliations:** ^1^Department of Pharmacy, Medical College, Henan University of Science and Technology, Luoyang, China; ^2^Department of Pharmacology, School of Basic Medicine, Tongji Medical College, Huazhong University of Science and Technology, Wuhan, China; ^3^Medical College, Henan University of Science and Technology, Luoyang, China

## Abstract

**Objective:**

Obesity causes a variety of metabolic alterations that may contribute to abnormalities of the cardiac structure and function (obesity cardiomyopathy). In previous works, we have shown that pentamethylquercetin (PMQ) significantly improved metabolic disorders in obese mice and it inhibited pressure overload-induced cardiac remodeling in mice. However, its potential benefit in obesity cardiomyopathy remains unclear. The aim of this study was to investigate the effects of PMQ on cardiac remodeling in obese mice.

**Methods:**

We generated a monosodium glutamate-induced obese (MSG-IO) model in mice, which were treated with PMQ (5, 10, and 20 mg/kg) for 16 weeks consecutively. We examined the metabolic parameters and observed cardiac remodeling by performing cardiac echocardiography and Masson's staining. The expression levels of molecules associated with the endogenous antioxidant system, including the sestrins/kelch-like ECH-associated protein 1 (Keap1)/Nuclear factor (erythroid-derived 2)-like 2 (Nrf2) signaling pathway, were analyzed by western blotting and immunofluorescent staining.

**Results:**

We found that PMQ treatment significantly ameliorated obesity phenotypes and improved metabolic disorders in MSG-IO mice. PMQ decreased the heart wall thickness and attenuated cardiac fibrosis. Further study revealed that the protective effects of PMQ might be mediated by promoting Keap1 degradation and augmenting sestrins expression and Nrf2 nuclear translocation.

**Conclusion:**

Our findings indicated that PMQ ameliorated cardiac remodeling in obese mice by targeting the sestrins/Keap1/Nrf2 signaling pathway.

## 1. Introduction

Obesity can cause a variety of alterations that may predispose to changes in the cardiac morphology and ventricular function [1, 2]. Several mechanisms are associated with obesity cardiomyopathy, including inflammation and various neurohormonal and metabolic abnormalities. Oxidative stress, another important factor, plays a significant role in myocardial abnormalities related to obesity [3].

Sestrins, a family of highly conserved stress-inducible proteins, are considered to be important components of antioxidant defense [3]. Genetic depletion of sestrins has been found to result in exacerbation of obesity-associated pathological disorders, including fat accumulation, insulin resistance, mitochondrial pathologies, and cardiac dysfunction, in multiple animal models, which could be relieved by treatments that suppress ROS signaling [4]. Current studies assessing the direct role of sestrins in cardiac pathophysiology are relatively limited. Our previous study showed that Sestrin2 participated in the cardiac remodeling process, and the pentamethylquercetin (PMQ) protective effect against cardiac remodeling induced by pressure overload was sestrin2 dependent [5]. However, whether sestrins have a protective effect on obesity cardiomyopathy needs to be studied further.

Nuclear factor (erythroid-derived 2)-like 2 (Nrf2) is the master regulator of oxidative stress signaling. In normal environments, kelch-like ECH-associated protein 1 (Keap1) binds to Nrf2 for proteasomal degradation. Under oxidative stress circumstances, Keap1 inactivation occurs through oxidation of its several cysteine residues or by autophagic elimination mediated by an autophagy adapter p62/sequestosome-1 (SQSTM1) [6]; then, Nrf2 is released from this complex, and it enters into the nucleus to promote antioxidant gene expression. Nuclear Nrf2 can upregulate the expressions of a wide range of antioxidant genes, such as Glutathione-S-transferase (GST), HMOX1 (HO-1), NQO1, and others by binding with the antioxidant response element (ARE) [7]. Sestrins are transcriptionally induced through diverse transcription factors including Nrf2 under oxidative stress [8], and then sestrin2 promotes ULK1-induced phosphorylation of SQSTM1, which further facilitates Keap1 degradation and Nrf2 activation and nuclear translocation in hepatocytes. Indeed, the sestrins/Keap1/Nrf2 pathway is physiologically important for the antioxidant defense of hepatocytes [6], but whether it plays a key role in cardiac remodeling of obese mice has not yet been reported.

PMQ, identified as a typical member of the polymethoxylated flavones (PMF), is widely distributed in a variety of vegetables, fruits, and Chinese herbal medicines. PMQ has also been proven to improve the pressure overload and angiotensin II-induced cardiac remodeling in rats and mice [9, 10]. Nevertheless, there is no report about whether PMQ can improve obesity-related cardiac remodeling. Therefore, the present study investigated the protective effects of PMQ on cardiac remodeling in monosodium glutamate-induced obese (MSG-IO) mice and ascertained whether the protective effects of PMQ are associated with the sestrins/Keap1/Nrf2 signaling pathway.

## 2. Materials and Methods

### 2.1. Reagents

PMQ was synthesized by the Food and Drug Evaluation Center of Tongji Medical College at Huazhong University of Science and Technology at a purity of 99.5%, as examined by HPLC. MSG was purchased from Sigma (USA). Metformin was obtained from Tianjin Biochemical Pharmaceutical Factory (Tianjin, China). Rabbit polyclonal antibodies specific for sestrin1, sestrin2, Nrf2, Keap1, HMOX-1 (HO-1), and Lamin B1 were purchased from Proteintech (Wuhan, China). Mouse polyclonal antibodies specific for GAPDH were acquired from Boster (Wuhan, China). All materials for SDS-PAGE were obtained from Biyuntian or Guge (China).

### 2.2. Animal Care

CD1 male and female mice at 10 weeks of age were purchased from the Medical Experimental Animal Center of Huazhong University of Science and Technology, China. After being fed adaptively for 1 week, virgin female mice were mated with male mice at a ratio of 4 : 1 to get newborn mice. The animals were housed in a temperature- and humidity-regulated room (22 ± 2°C and 50 ± 5%, respectively) with controlled lighting (12-h light/dark cycle). Food and water were freely available. All experimental protocols were approved by the Animal Care and Ethics Committee of Huazhong University of Science and Technology (SCXK2017-0010), and they were in accordance with the Guide for the Care and Use of Laboratory Animals of National Institutes of Health Guidelines.

### 2.3. Establishment of the Metabolic Syndrome Model and Experimental Groups

From days 2 to 8 after delivery, newborn mice were given a solution of MSG dissolved in saline (3 mg/g) or equivalent saline subcutaneously in their back via a microsyringe [11]. At 8 weeks of age after being fed routinely, all treated male mice were randomly (method of random numbers table) divided into the following six groups (*n* = 12 per group): control; MSG; PMQ 5, 10, and 20 mg/kg; and metformin 300 mg/kg. The PMQ groups were given medicated feed with different drug concentrations for 16 weeks, and the metformin group was administered the drug in drinking water. Body weight, body length, waistline, and food/water consumption were monitored weekly, and the Lee index was calculated (dividing the cubic root of body weight by the body length) at the end of the experiment.

### 2.4. Metabolic Measurements

At 18 weeks of age, blood was collected from the tail artery of mice for plasma glucose evaluation by using a blood glucose tester (Roche, Germany) after 12 h fasting, and after that, an oral glucose tolerance test (OGTT, 0.2 g/kg) was also performed. Simply, blood samples were collected through the tail artery at 5, 15, 30, 60, and 120 min after the administration of oral glucose by gavage, and the plasma glucose concentration was tested as mentioned above. After the evaluation, the animals were continuously fed for recovery and given medicated fodder with different drug concentrations accordingly.

### 2.5. Assessment of the Cardiac Structure and Function: Echocardiography

Two-dimensional-guided M-mode echocardiography was performed in mice at 23 weeks of age using the Vevo 1100 imaging system equipped with a 30-MHz high-frequency linear transducer (Visual Sonics, Toronto, Canada). Briefly, mice were lightly sedated with chloral hydrate (4%, intraperitoneal injection), and the prethoracic skin hair was shaved using a commercially available depilatory cream. A guided M-mode echocardiogram was recorded through the anterior and posterior left ventricular (LV) walls at 21 frames/s. Images were obtained at the level of the papillary muscle tips, and then measurements were performed to obtain the LV anterior wall at diastole (LVAW, d) and systole (LVAW, s), the LV posterior wall at diastole (LVPW, d) and systole (LVPW, s), the LV internal dimension at diastole (LVID, d) and systole (LVID, s), and the interventricular septum thickness at diastole (IVS, d) and systole (IVS, s) in M-mode. Percentage fractional shortening (FS%) and ejection fraction (EF%) were calculated via the Visual Sonics Measurement Software.

### 2.6. Serum Analysis and Cardiac Weight Index Calculation

At 24 weeks of age after completing the echocardiography record, mice were weighed. Euthanasia was performed under deep anesthesia (sodium pentobarbital by intraperitoneal injection at the dose of 45 mg/kg) by cervical dislocation, and all efforts were made to ameliorate suffering. Blood was collected from the orbital venous sinus for fasting serum glucose and insulin testing in each group, which were performed using the corresponding commercial kits (Biosino, Beijing, China). The HOMA of insulin resistance (HOMA-IR) was calculated and used to assess insulin resistance [12]. The hearts were rapidly removed and washed in 0.9% ice-cold saline, the surface fluid was sucked out with a filter paper, and they were weighed. The heart weight/tibial length (HW/TL) and the left ventricular weight/tibial length (LHW/TL) were calculated. Then, part of the LV samples was flash-frozen in liquid nitrogen and stored at −80°C until further molecular biology experimentation, and the other LV samples were fixed in 4% paraformaldehyde for 24 h and then embedded in paraffin for subsequent morphology detection.

### 2.7. Histological Examination of Interstitial Fibrosis

Sections were cut longitudinally at 5 *μ*m thickness. Interstitial fibrosis in the left ventricle was determined using Masson's trichrome staining. To assess the degree of fibrosis, the images were quantitatively analyzed by morphometry with Image-Pro Plus software (NIH, 1.61).

### 2.8. Determination of Nucleus Nrf2 Using the Immunofluorescence Staining Method

The paraffin samples (5 *μ*m) were removed from the sections with xylene, rehydrated in graded alcohol series, subjected to antigen retrieval in EDTA buffer (pH 8.0, Goodbio technology) using a microwave, and then placed in 3% BSA to block nonspecific staining for 30 min at room temperature. After that, the sections were incubated with anti-Nrf2 antibody (1 : 100, Goodbio technology) at 4°C overnight, followed by the fluorescent-labeled secondary antibody at lucifuge and room temperature for 50 min (1 : 300; Goodbio technology). After counterstaining with DAPI haematoxylin, the sections were dehydrated and viewed under a fluorescence microscope (400× amplification; Nikon). The ultraviolet excitation wavelength was 330–380 nm, the emission wavelength was 420 nm, the CY3 red light excitation wavelength was 510–560 mm, and the emission wavelength was 590 nm.

### 2.9. Western Blot Analysis

Total proteins were extracted from the left ventricle with ice-cold lysis buffer (RIPA). Nuclear proteins were extracted from the left ventricle with the nucleoprotein extraction kit. The quantity of protein was measured by the BCA method. Denatured protein samples (30 *μ*g) were subjected to SDS-PAGE. After electrophoresis, protein was transferred to PVDF membranes, which were then blocked for 1 h by 5% nonfat dry milk in TBS-T (mM, Tris-base 24.8, NaCl 136.8, KCl 2.7, 0.1% Tween-20, pH 7.4) and subsequently incubated with primary antibody in 5% nonfat dry milk at 4°C overnight. After washing thrice with TBS-T, the membranes were incubated with anti-rabbit or anti-mouse horseradish peroxidase-conjugated secondary antibody (1 : 5000 dilutions) for 1 h. The signal was detected by chemiluminescence using the ECL detection system (Amersham). The same procedure was repeated for Nrf2 (1 : 500), sestrin1/2 (1 : 500), Keap1 (1 : 500), HO-1 (1 : 500), Lamin B1 (1 : 5000), and GAPDH (1 : 8000). Lamin B1 and GAPDH were used as loading controls for nucleoprotein and total protein. Quantification of bands was performed using Image J Software (NIH).

### 2.10. Statistical Analyses

All values were expressed as mean ± SEM. Differences among groups were evaluated using the one-way ANOVA test. *P* < 0.05 was considered statistically significant.

## 3. Results

### 3.1. PMQ Attenuated Obesity and Metabolic Disorders in MSG-IO Mice

**Figure 1:**
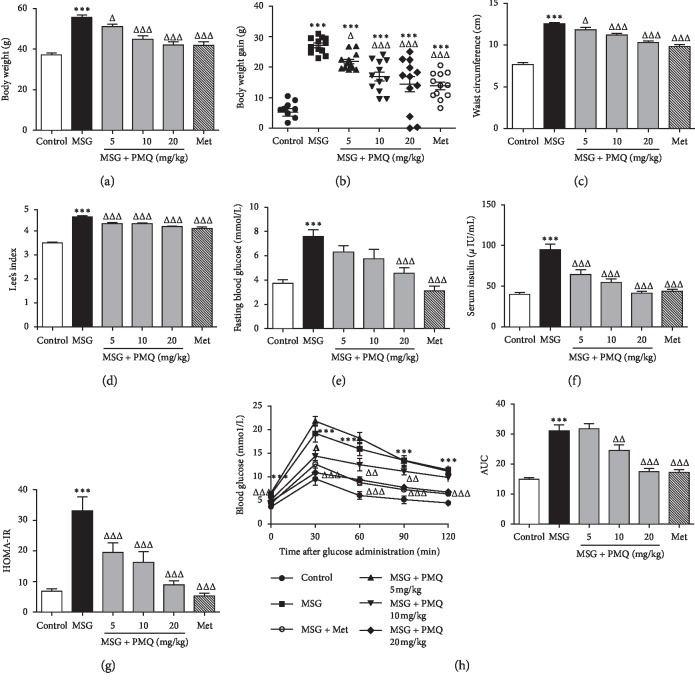
PMQ ameliorated obesity and metabolic disorders in MSG-IO mice at the 24th week. PMQ treatment resulted in significant reductions in (a) body weight, (b) body weight gain, (c) waist circumference, and (d) LEE index. Hyperglycemia (e), hyperinsulinaemia (f), and HOMA-IR (g) were significantly improved after PMQ treatment compared with the corresponding MSG-IO mice. (h) Left: effect of PMQ on the glucose tolerance disorder, as measured by the oral glucose tolerance test (OGTT). Right: area under the curve (AUC). Data are expressed as mean ± SEM, *n* = 12. ^*∗∗∗*^*P* < 0.001 vs. control group; ^Δ^*P* < 0.05, ^ΔΔ^*P* < 0.01, ^ΔΔΔ^*P* < 0.001 vs. MSG group.

For further confirmation of our previous results, we examined the effects of PMQ on metabolic disorders in MSG-IO mice. As shown in Figure 1, PMQ significantly reduced the body weight and body weight gain (Figures 1(a) and 1(b)), waist circumference (Figure 1(c), and the LEE index (Figure 1(d)); lowered fasting blood glucose (Figure 1(e)) and serum insulin levels (Figure 1(f)); and ameliorated insulin resistance in MSG-IO mice (Figure 1(g)). PMQ also improved glucose tolerance (Figure 1(h)). Our results further confirmed that PMQ dose-dependently improved metabolic disorders in MSG-IO mice. We simultaneously observed that metformin 300 mg/kg produced a comparable benefit to PMQ 20 mg/kg.

### 3.2. PMQ Prevented Cardiac Hypertrophy in MSG-IO Mice

**Figure 2:**
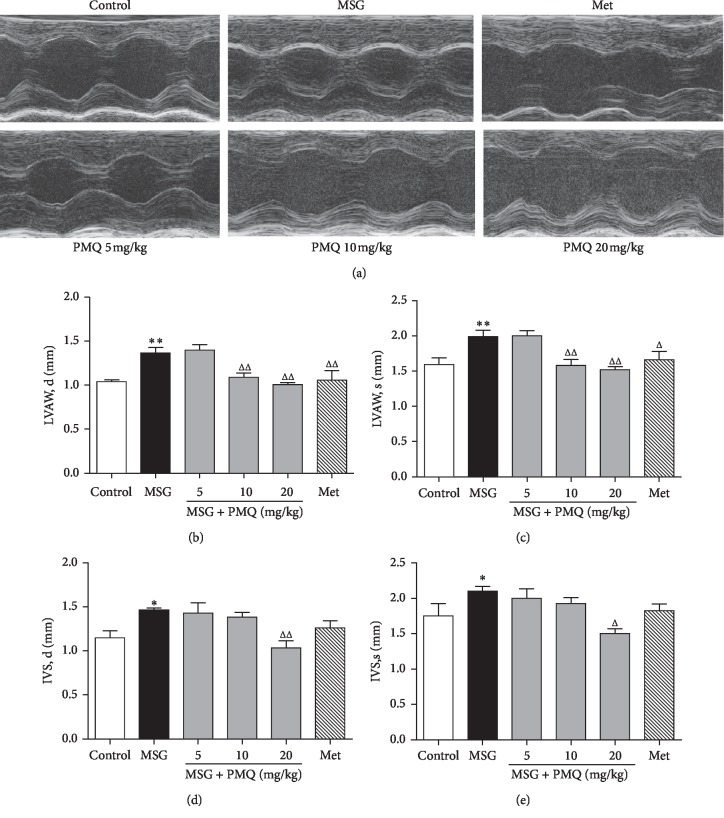
Echocardiographic measures of cardiac remodeling in MSG-IO mice at the end of 23 weeks. (a) Representative M-mode frames from the midpapillary region; (b) diastolic thickness of the LV anterior wall (LVAW; d); (c) systolic thickness of the LV anterior wall (LVAW; s); (d) diastolic thickness of the interventricular septum (IVS; d); and (e) systolic thickness of the interventricular septum (IVS; s). Data are expressed as mean ± SEM, *n* = 6 − 8. ^*∗*^*P* < 0.05, ^*∗∗*^*P* < 0.01 vs. control group; ^Δ^*P* < 0.05, ^ΔΔ^*P* < 0.01 vs. MSG group.

To investigate the effects of PMQ on obesity-related cardiac remodeling in mice, we evaluated the heart wall thickness and cardiac function by the echocardiography method at week 23.  Figure 2(a) shows the representative M-mode frames from different groups. EF%, FS%, and LVID were not significantly changed in MSG-IO mice compared with the control group, while LVAW and IVS at the end of systole and diastole phases were significantly increased in MSG-IO mice (*P* < 0.01 or *P* < 0.05) (Figures 2(b)−2(e)). In the 10 and 20 mg/kg treatment groups, PMQ was able to attenuate the increase in LVAW (*P* < 0.01) (Figures 2(b) and 2(c)); however, only PMQ 20 mg/kg exerted statistically significant effects on IVS, d (*P* < 0.01) (Figure 2(d)) and IVS, s (*P* < 0.05) (Figure 2(e)). Metformin could prevent obesity-related cardiac hypertrophy to the same extent as PMQ 10 mg/kg.

### 3.3. PMQ Reduced the Cardiac Weight Index and Ameliorated Cardiac Fibrosis in MSG-IO Mice

**Figure 3:**
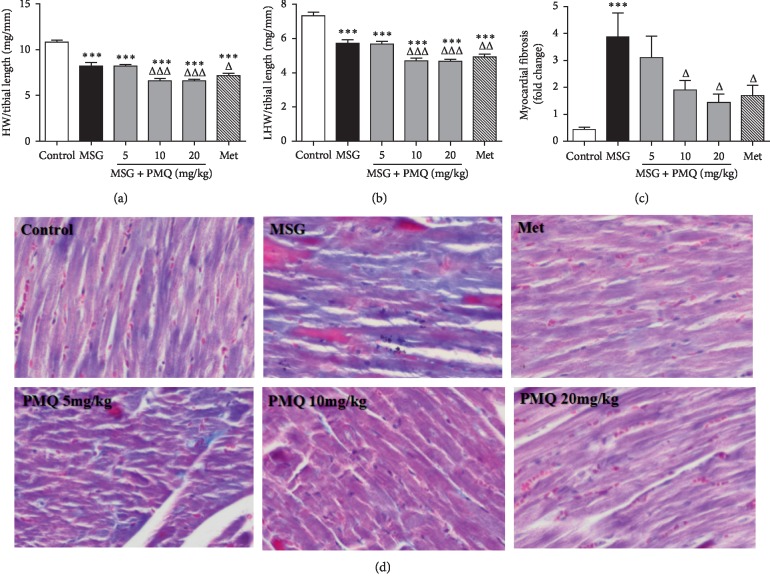
PMQ reduced the cardiac weight index and ameliorated cardiac fibrosis in MSG-IO mice at the end of the 24th week. (a) Cardiac weight index and (b) left ventricular weight index were calculated. (c) Statistical results of myocardial fibrosis of (d); and (d) Representative figures of interstitial fibrosis (Masson's trichrome staining ×200). Data are expressed as mean ± SEM, *n* = 6. ^*∗∗∗*^*P* < 0.001 vs. control group; ^Δ^*P* < 0.05, ^ΔΔ^*P* < 0.01, ^ΔΔΔ^*P* < 0.001 vs. MSG group.

To determine whether PMQ could inhibit cardiac fibrosis, we evaluated the heart weight index and morphology to assess cardiac remodeling in MSG-IO mice. The heart size was decreased, while the heart wall was stiff in MSG-IO mice. The heart weight index or the LV weight index was also significantly decreased compared with that in the control group (*P* < 0.001) (Figures 3(a) and 3(b)). However, the area of cardiac fibrosis was significantly increased (Figures 3(c) and 3(d)), indicating the development of cardiac remodeling in MSG-IO mice. PMQ treatment reduced the heart weight index, inhibited cardiac fibrosis, and improved heart wall stiffness. Metformin 300 mg/kg antagonized cardiac remodeling in MSG-IO mice to the same extent as PMQ 10 mg/kg.

### 3.4. PMQ Enhanced Sestrins Expression and Keap1 Degradation in the Hearts of MSG-IO Mice

**Figure 4:**
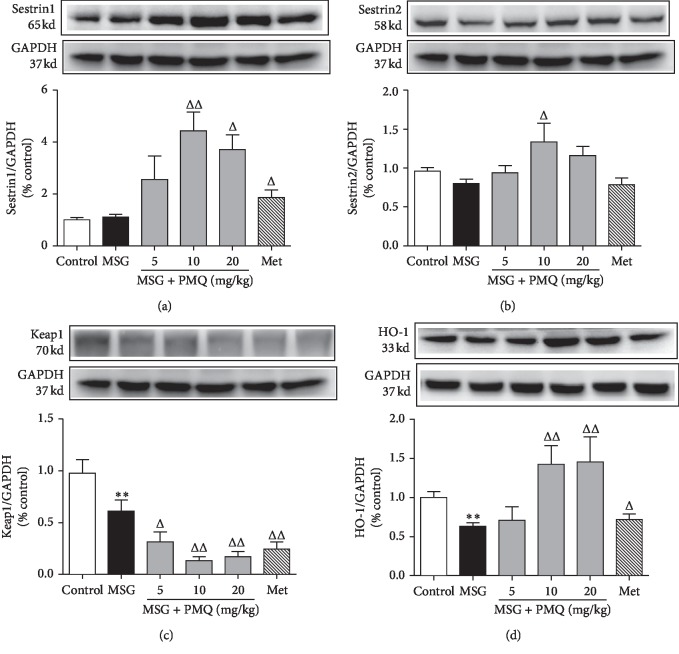
Effect of PMQ on the protein levels of sestrins, Keap1, and Nrf2 downstream targets HO-1 in the cardiac tissue of MSG-IO mice. (a) Representative immunoblot and relative density analysis of sestrin1; (b) representative immunoblot and relative density analysis of sestrin2; (c) representative immunoblot and relative density analysis of Keap1; and (d) representative immunoblot and relative density analysis of HO-1. The relative density was expressed as the ratio (sestrin1/2, Keap1, and HO-1/GAPDH). Data are expressed as mean ± SEM, *n* = 6. ^*∗∗∗*^*P* < 0.001 vs. control group; ^Δ^*P* < 0.05, ^ΔΔ^*P* < 0.01 vs. MSG group.

To understand the molecular mechanisms of PMQ-mediated improvement of cardiac remodeling in obese mice, we investigated the protein expressions associated with the endogenous antioxidant system. At the age of 24 weeks, the protein levels of sestrin1, sestrin2, and Keap1 showed a slight increase or decrease (Figures 4(a)−4(c)). However, the level of antioxidant protein HO-1, the downstream target gene of Nrf2, was significantly decreased in the heart tissue of MSG-IO mice (Figure 4(d)). As expected, it was observed that PMQ significantly reduced Keap1 expression in a dose-dependent manner (*P* < 0.05, *P* < 0.01), and it dramatically upregulated sestrin1, sestrin2, and HO-1 expressions in the heart tissue of obese mice (*P* < 0.05, *P* < 0.01). Metformin 300 mg/kg served as a positive control, and it increased sestrin1 and HO-1 expressions and decreased Keap1 expression, and the alterant degree was akin to PMQ 5 mg/kg.

### 3.5. PMQ Promoted Nrf2 Translocation into the Nucleus in the Heart Tissue of MSG-IO Mice

**Figure 5:**
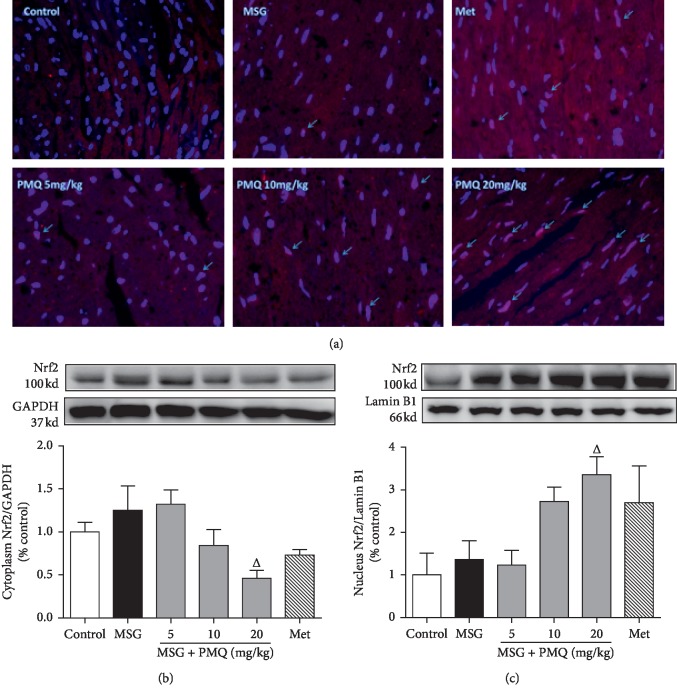
PMQ promoted the endogenous antioxidant system by boosting Nrf2 nuclear translocation in the hearts of MSG-IO mice. (a) Representative images for immunofluorescent staining of Nrf2 nuclear translocation in the formalin-fixed myocardial tissues (×200). Western blot analysis for the protein expression of Nrf2 in the cytoplasm (b) and the nucleus (c). Data are expressed as mean ± SEM, *n* = 6. ^Δ^*P* < 0.05 vs. MSG group.

Figure 5(a) shows the representative images of Nrf2 expression in the nucleus by immunofluorescent staining, in which Nrf2 showed a slight increase in the heart tissue of obese mice. PMQ induced a significant increase in nuclear Nrf2 and a significant decrease in the cytoplasmic Nrf2 protein expression (*P* < 0.05) in the PMQ 20 mg/kg, indicating that PMQ treatment may activate Nrf2 and induce Nrf2 nuclear translocation (Figures 5(b) and 5(c)). Furthermore, metformin, as a positive control, promoted Nrf2 nuclear translocation at the same extent as PMQ 10 mg/kg.

## 4. Discussion

In this study, we showed that PMQ could resist cardiac remodeling associated with obesity, which might be mediated by improving the metabolism disorders and enhancing the endogenous antioxidant system of the sestrins/Keap1/Nrf2 signaling pathway. These findings suggest the potential of sestrins as the key molecules and drug targets for obesity-related cardiac remodeling.

Dietetic intervention, such as high-fat diet, would be associated with the manifestation of obesity, metabolic disturbances, including glycemic and lipid disorders, and evidence of cardiac hypertrophy and interstitial fibrosis [13]. An obesity model can also be acquired by pharmacological induction in rodent species [14–16]. The administration of MSG to newborn rodents provokes hypothalamic injury and results in several neuroendocrine and metabolic abnormalities, including obesity, hypoactivity, delayed puberty, and elevated plasma corticosterone levels [17]. The MSG-IO model exhibits most of the features observed in human obesity, such as abdominal obesity, hyperinsulinemia, and insulin resistance. Moreover, interstitial and perivascular collagen density and increased media-to-lumen ratio of intramyocardial arterioles were observed in MSG-IO rats [14], indicating that the initial process of cardiac remodeling is associated with obesity and/or insulin resistance. Few studies have evaluated the cardiac changes in the MSG-IO mouse model.

Our results indicated that at the age of 24 weeks, MSG-IO mice showed apparent metabolic syndrome appearances; increased body weight, body weight gain, waist circumference, and LEE index; and significant increases in fasting blood glucose and serum insulin levels and HOMA-IR index. All these pathological changes could be mitigated by PMQ treatment, and PMQ also improved glucose tolerance, which was consistent with our previous findings [11]. Ventricular wall displayed thickening on echocardiography in MSG-IO mice. Masson's staining of the heart tissue confirmed the existence of myocardial fibrosis in MSG-IO mice. PMQ significantly ameliorated ventricular wall stiffness and myocardial remodeling, as proved by echocardiography and Masson's staining. At the same time, it was noted that the hearts were smaller and stiffer in MSG-IO mice compared with the control group, and the cardiac weight index was similarly reduced. Generally, in a high-fat diet-induced obese animal model, it appeared that cardiac hypertrophy occurred concurrently with an increase in the heart weight index. This controversial finding might be due to the use of different modeling methods and different disease stages, while in the MSG-IO model, MSG was given from the next day of birth, in which MSG entered the central nervous system and may bring in a series of negative impacts on animal development, such as neuroendocrine abnormalities. At the same time, obesity began at preadolescence, which may cause irreversible effects on the development of the heart, although it is still not clear. Studies on the effects of preadolescent obesity on the body are gaining more and more attention.

Recent studies have implicated oxidative stress in the pathophysiology of obesity-related cardiovascular disorder [18]. PMQ has been proven to improve the metabolic disorders in the body and attenuate high-fat diet-induced visceral adipogenesis via stimulation of AMP-dependent protein kinase (AMPK) activity and suppression of the Sirt1-mediated mTOR and adipogenesis signaling cascades [11, 19] and to inhibit cardiac hypertrophy responses in vivo and in vitro involving PPAR *α* and PPAR *β* upregulation [9, 10], suggesting that PMQ has a protective effect on metabolic syndrome and cardiovascular diseases. However, a better understanding of the possible mechanisms involved in this process is still required. The Keap1/Nrf2 pathway is physiologically important for the antioxidant defense, and it serves as a negative regulator of cardiac remodeling and dysfunction [20]. Sestrins, known as the important components of antioxidant defense, have been proved to improve the metabolic function in the liver and prevent age-related pathologies by suppressing the mammalian target of rapamycin complex 1 (mTORC1) activity through the activation of AMPK [21, 22]. Sestrin2 can protect the heart from ischemia reperfusion injury by activating AMPK signaling [23]. Sestrin2 counter regulates renal ROS production and may contribute to the maintenance of normal blood pressure [24]. However, whether sestrins can provide cardiovascular protection through upregulating the Keap1/Nrf2 pathway in obese mice is still unknown.

The results of our study showed that the expression of Nrf2 in the heart probably acts as a compensatory mechanism toward oxidative stress in MSG-IO mice. Meanwhile, Keap1 and sestrin2 expressions were slightly decreased, while sestrin1 expression was gently increased. As shown in Figure 4, PMQ potently increased sestrin1/2 expression; especially, it significantly increased sestrin1 expression and dramatically decreased Keap1 expression, which might have activated Nrf2 and elevated the nuclear translocation, as illustrated in Figure 5, which upregulated the expression of its downstream antioxidant enzymes such as HO-1. We also examined the level of Keap1 mRNA. As shown in Supplementary [Supplementary-material supplementary-material-1], there was no significant difference among the different groups, that suggests PMQ acts mainly by promoting the protein degradation, not by inhibiting the Keap1 transcription process. Together, these data suggested that PMQ has protective effects on obesity-related cardiac remodeling, which could be potentially due to its effect of improving the metabolic disorders and enhancing the endogenous antioxidant function. Thus, PMQ could upregulate the expression of sestrin1/2 and then accelerate Keap1 degradation, thus further activating Nrf2 signaling. In turn, maybe sestrins are targets of Nrf2, which activate and then promote the expression of downstream target genes such as HO-1 and sestrin2. We speculated sestrins and Nrf2, constituting a positive feedback loop, while further investigations need to be conducted in the future.

Metformin is currently being used as the first-line pharmacological agent for type 2 diabetes. Besides the effect of metformin in improving the metabolism, there is growing interest in its effects on cardiovascular diseases [25–27], cancer [28], and aging [29]. Metformin-induced activation of the energy-sensor AMPK is well documented, while AMPK-independent mechanisms are also recorded, including the inhibitory action on TGF-*β*1 expression [30], suppression of ROS generation via the inhibition of the NADPH oxidase pathway [31], and eNOS activation [32]. Pretreatment with metformin activates the Nrf2 antioxidant pathways in the hippocampus of patients with global cerebral ischemia [33]. In this study, metformin served as a positive control; the results showed that metformin prevented cardiac remodeling in MSG-IO mice, this effect was similar to that of PMQ 10 mg/kg, and the mechanism was partly associated with enhancing the endogenous antioxidant system through sestrin1/Keap1/Nrf2 signaling.

We chose chloral hydrate while performing echocardiography of the heart, and the aim was to mainly calm the animals with ease to operate, as it was a noninvasive test and took very little time, less than 1 min per animal. After finishing the test, the animals wake up very quickly, which can minimize the incidence of accidents.

However, further understanding of sestrins antioxidant signal transduction is necessary for the development of novel therapeutic strategies against cardiac remodeling, and more studies are needed to elucidate the direct targets of PMQ in regulating the sestrins signaling pathway. In the future study, we will conduct sestrin 1/2 structure docking with PMQ and molecular dynamics simulation to ascertain if there is a direct interaction between them. Furthermore, our previous study also found that PMQ has an effect on cardiac electrophysiology, and if PMQ resisting cardiac remodeling is associated with ion channels on cell membrane, especially calcium channels, is worth studying, and we are going to do further study about this.

## 5. Conclusions

Metabolic disorders and adverse cardiac remodeling were significantly evident in MSG-IO mice. PMQ exhibited potent protective effects on metabolic disorders and cardiac remodeling, which were associated with its effect of enhancing the endogenous antioxidant function through the activation of sestrins/Keap1/Nrf2 signaling. Our study strongly indicated that sestrins might be therapeutic targets, while PMQ could be an effective treatment option for obesity-related cardiac remodeling.

## Data Availability

All data of this study can be acquired from the author if necessary.

## Supplementary Materials

Supplementary Materials 1.Arrive checklist, 2.Original unadjusted western Blots, and 3.SUPPL. FIGURE 1: effect of PMQ on the mRNA levels of Keap1 in the cardiac tissue of MSG-IO mice.
